# Temperature-Induced Structural Phase Transitions in Nano-Fe-Modified Sustainable Biochar for Efficient Sb(III) Adsorption

**DOI:** 10.3390/nano16201288

**Published:** 2026-10-11

**Authors:** Lei Wang, Bo Song, Qi Wang, Gaoyani Ku, Chen Chen, Zhibin Wang, Jafar Ali, Evgeni M. Glebov, Xuliang Zhuang

**Affiliations:** 1 Laboratory of Environmental Biotechnology, Research Center for Eco-Environmental Sciences, Chinese Academy of Sciences, Beijing 100085, China; 2 College of Resources and Environment, University of Chinese Academy of Sciences, Beijing 100049, China; 3 Institute of Fungus Resources, Key Laboratory of Plant Resource Conservation and Germplasm Innovation in Mountainous Region (Ministry of Education), College of Life Sciences, Guizhou University, Guiyang 550025, China; 4 Henan Institute of Advanced Technology, Zhengzhou University, Zhengzhou 450003, China; 5 Hubei Key Laboratory for Processing and Transformation of Agricultural Products, College of Food Science & Engineering, Wuhan Polytechnic University, Wuhan 430023, China; 6 Institute of Sustainable Energy (ISE), Universiti Tenaga Nasional (UNITEN), Kajang 43000, Malaysia; 7 Department of Water Quality Management, Karlsruhe Institute of Technology (KIT), 76131 Karlsruhe, Germany; 8 Voevodsky Institute of Chemical Kinetics and Combustion, Siberian Branch of the Russian Academy of Sciences, 3 Institutskaya Str., 630090 Novosibirsk, Russia; 9 Novosibirsk State University, 2 Pirogova Str., 630090 Novosibirsk, Russia; 10 State Key Laboratory of Tibetan Plateau Earth System, Environment and Resources (TPESER), Institute of Tibetan Plateau Research, Chinese Academy of Sciences, Beijing 100101, China

**Keywords:** iron-modified biochar, pyrolysis temperature, iron phase transitions, antimony(III), adsorption, graphitization, antimony-containing wastewater

## Abstract

Nano-iron-modified biochar (Fe-SC) has emerged as a promising adsorbent for pollutant removal. However, how pyrolysis temperature changes its iron phases and affects Sb(III) removal remains unclear. In this work, Fe-SC was prepared at three pyrolysis temperatures (400 °C, 600 °C, and 800 °C) to investigate how structural phase transitions affect Sb(III) adsorption performance. XRD analysis suggests a possible evolution of Fe phases with increasing temperature, from an Fe_3_O_4_ crystalline phase at 400 °C to an Fe crystalline phase at 600 °C, and subsequently to an Fe_3_C crystalline phase with pronounced graphitization at 800 °C. These structural changes, together with the specific surface area and graphitization degree, strongly influenced Sb(III) adsorption. Fe-SC600 had the highest specific surface area (217.73 m^2^/g) and achieved nearly 80% Sb(III) removal, the highest among the three materials. Fe-SC also maintained effective Sb(III) adsorption over a broad pH range (3.0–9.0), and the presence of S^2−^ and HCO_3_^−^ ions enhanced Sb(III) removal under the tested conditions. These findings highlight the importance of optimizing pyrolysis conditions to enhance the adsorption performance of Fe-SC, with Fe-SC600 demonstrating the most promising balance of structural properties and adsorption efficiency for environmental remediation applications.

## 1. Introduction

Metal-modified biochar materials have attracted significant attention in environmental remediation because of their enhanced ability to remove pollutants compared to unmodified biochar [1,2]. Biochar is produced by pyrolyzing biomass, which is valued for its large surface area, porosity, and abundance of surface functional groups [3,4], but its pollutant removal efficiency is limited by its surface chemistry [5,6]. Adding active metal species to biochar matrices presents a promising strategy to overcome these limitations [7,8]. Metal-modified biochar exhibited improved adsorption capacity, increased chemical reactivity, and enhanced catalytic properties [2,9], which collectively contribute to more effective pollutant capture and degradation.

Among various metal-modified biochars, nano-iron-modified biochar (Fe-SC) materials are particularly notable for their unique properties and wide applicability in environmental remediation [10,11,12]. Iron in different oxidation states can interact with a wide range of pollutants, including heavy metals, organic contaminants, and metalloids [13,14,15,16]. The presence of iron on the biochar surface facilitated redox reactions, enhanced the generation of reactive oxygen species, and provided active sites for adsorption, improving its pollutant removal performance [17,18,19,20,21]. Fe-SC materials have been widely employed for the adsorption of antimony (Sb) [11,22,23], a toxic metalloid that poses serious environmental and health risks. Antimony is widely used in various industries such as flame retardants, plastics, paints, ceramics, ammunition, and lead-acid batteries [24]. Sb occurs primarily in trivalent (Sb(III)) and pentavalent (Sb(V)) forms, with Sb(III) being approximately ten times more toxic than Sb(V) [16,25,26]. Due to its high toxicity, antimony and its compounds have been designated as a priority pollutant by both the United States Environmental Protection Agency (US EPA) and the European Environment Agency (EEA) [16,27], underscoring the urgency of effective removal strategies [28]. A crucial factor in the effectiveness of Fe-SC is the temperature-induced structural phase transitions during preparation. These phase transitions can significantly modify the material’s crystallinity, pore structure, and surface chemistry, thereby affecting its pollutant adsorption. However, previous studies have primarily focused on the impact of preparation temperatures on the adsorption properties of the material [22,29,30], and there has been limited attention to the temperature-induced structural phase transitions of Fe-SC. Therefore, a deeper understanding of these phase transitions is essential to identifying the active phases responsible for pollutant removal and optimizing the performance of materials in environmental applications.

In this study, Fe-SC was systematically prepared at three distinct pyrolysis temperatures (400 °C, 600 °C, and 800 °C) to investigate the influence of these temperature-induced structural phase transitions on Sb(III) removal from wastewater. By characterizing the structural phase transitions and adsorption properties of Fe-SC at each temperature, this work aimed to elucidate the relationship between phase transitions and adsorption properties. This study examined how different pyrolysis conditions affect the performance of Fe-SC and offered valuable insights for optimizing biochar preparation to enhance its application in environmental remediation.

## 2. Materials and Methods

### 2.1. Materials

Sugarcane bagasse was obtained from Guangxi province, China. Potassium antimony tartrate (C_8_H_4_K_2_O_12_Sb_2_·3H_2_O, 98%) was provided by Macklin Bio-Chem Technology Co., Ltd. (Shanghai, China). Sodium hydroxide (NaOH) and hydrochloric acid (HCl) of analytical grade were purchased from Aladdin Industries (Shanghai, China). Ferric citrate (FeC_6_H_5_O_7_) of analytical grade was purchased from China National Pharmaceutical Group Chemical Reagent Co., Ltd. The stock solution of Sb(III) (200 mg·L^−1^) was prepared by dissolving C_8_H_4_K_2_O_12_Sb_2_·3H_2_O in deionized water at room temperature. A standard solution containing 1000 mg/L of Sb(III) was obtained from the National Research Center for Certified Reference Materials of China.

### 2.2. Preparation of Fe-SC

**Figure 1:**
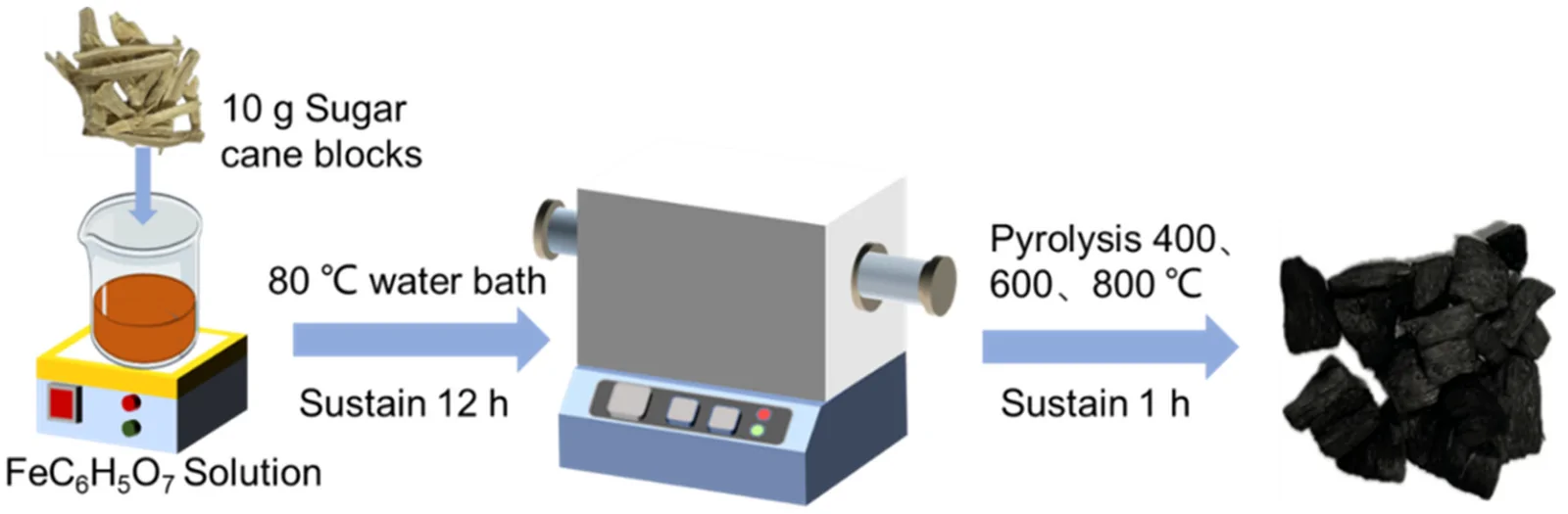
Preparation of iron-loaded porous biochar by pyrolysis.

The synthesis route of Fe-SC using sugarcane and Ferric citrate(FeC_6_H_5_O_7_) as raw materials is illustrated in Figure 1. Sugarcane was dried overnight in an oven at 60 °C. Approximately 10 g of dried sugarcane chunks were crushed into sugarcane bagasse. The bagasse was added to 100 mL of a 12.2 g/L FeC_6_H_5_O_7_ solution in an 80 °C water bath for 12 h and then dried overnight in an oven at 60 °C. The amount of FeC_6_H_5_O_7_ was 1.22 g, corresponding to a FeC_6_H_5_O_7_-to-biomass mass ratio of 0.122:1. Based on the FeC_6_H_5_O_7_ formula, the theoretical Fe-to-biomass mass ratio was approximately 0.0278:1. This value represents the feed ratio and does not denote the Fe loading in the final adsorbent. The treated bagasse was heated in a high-temperature muffle furnace at 400, 600, and 800 °C for 1 h under N_2_. The heating rate was 10 °C/min from room temperature to the target temperature. The N_2_ flow rate was not quantitatively controlled by a mass flow controller. In practice, N_2_ was continuously introduced and manually adjusted to maintain a steady gas outflow at the furnace outlet, thereby ensuring a slight positive pressure inside the furnace and preventing air backflow. After pyrolysis, the products were cooled and directly ground into powder without washing. The resulting biochars were named according to the pyrolysis temperature; i.e., the Fe-SC prepared at 400, 600, and 800 °C was denoted Fe-SC400, Fe-SC600, and Fe-SC800, respectively. The Fe-SC samples were stored in a closed container for subsequent experimental use. The biochar yield and total Fe content of the adsorbents were not determined in this study.

### 2.3. Batch Adsorption Experiment and Fitting Models

Batch adsorption experiments were conducted with the initial concentration of Sb(III) fixed at 20 μg/L. The effect of the dosage of Fe-SC (ranging from 1 g/L to 10 g/L) on the efficiency of removing Sb(III) from water was investigated through a preliminary adsorption test. After adsorption, all samples were filtered through a 0.45 μm polyethersulfone (PES) membrane (Shanghai Anpu Experimental Technology Co., Ltd., Shanghai, China), and the Sb(III) concentration in solution was determined using an inductively coupled plasma mass spectrometer (ICP-MS, Agilent 7800, Santa Clara, CA, USA). The Sb(III) removal rate was calculated using the following formula:

η = (C_0_ − C_t_)/C_0_ ×100% (1)

where C_0_ and C_t_ are the initial and final Sb(III) concentrations (μg/L).

All subsequent adsorption experiments were conducted in 50 mL beakers, each containing 0.24 g of sorbent and 40 mL of Sb(III) solution. The vessels were placed on a magnetic stirrer set at 300 rpm and 25 °C. To assess the adsorption isotherm of Fe-SC, 0.24 g of adsorbent was added to 40 mL of Sb(III) solutions with varying concentrations (20–200 μg/L). The Langmuir and Freundlich models were employed to fit the adsorption isotherm data. Kinetic adsorption experiments were carried out in a solution with an initial concentration of 200 μg/L Sb(III), with samples collected at specific time intervals ranging from 0 to 480 min. To evaluate the adsorption kinetic process, the Pseudo-first-order model, Pseudo-second-order model, Elovich model, and Intragranular diffusion model were selected to fit the kinetic data (Texts S1 and S2).

To investigate the impact of pH on Sb(III) adsorption, the initial concentration of Sb(III) was set at 20 μg/L, and the initial pH of the solution was adjusted to a range of 1.0–11.0. pH values were adjusted to 1, 3, 5, 7, 9, and 11 using either HCl or NaOH. The final Sb concentration in the filtrate was measured when the adsorption was at equilibrium. Sb(III) removal was calculated for each adsorbent at every tested pH.

To examine how coexisting substances affect Sb(III) removal by Fe-SC, we tested various substances including chloride (Cl^−^), nitrate (NO_3_^−^), sulfate (SO_4_^2−^), hydrogen phosphate (HPO_4_^2−^), fluoride (F^−^), sulfide (S^2−^), bicarbonate (HCO_3_^−^), humic acid (HA, natural dissolved organic compounds), and acetic acid (AA, low-molecular-weight organic acid). The anionic and natural organic matter content was set at 100 mmol/L.

All batch adsorption experiments were performed in triplicate. Statistical analyses were carried out using OriginPro 2022 software.

### 2.4. Characterization of Fe-SC

The surface morphology and main surface elements of Fe-SC were examined using field-emission scanning electron microscopy (FE-SEM, Hitachi SU8020, Tokyo, Japan) equipped with energy-dispersive X-ray spectroscopy (EDS). For sample preparation, a small amount of Fe-SC powder was attached to conductive carbon tape, and loosely adhering powder was blown off with N_2_. The crystal phases of Fe-SC were determined using an X-ray diffractometer (XRD, PANalytical X′ Pert3 Powder, Almelo, The Netherlands) with Cu-Kα radiation operated at 40 kV and 40 mA. The diffraction patterns were recorded over a 2θ range from 5° to 90°. For measurement, the Fe-SC powder was pressed flat into the sample holder to obtain a smooth surface. The specific surface area and porosity of the Fe-SC were measured using a Brunauer–Emmett–Teller (BET, Micromeritics ASAP 2460, Norcross, GA, USA) instrument. Before measurement, the samples were degassed under vacuum to remove adsorbed moisture and impurities. X-ray photoelectron spectroscopy (XPS, Thermo Scientific ESCALAB 250Xi, Waltham, MA, USA) with Al Kα radiation was performed to characterize the elemental composition of Fe-SC before and after Sb(III) adsorption. The binding energies were calibrated using the adventitious carbon C 1s peak at 284.8 eV. The obtained XPS spectra were fitted using the Advantage software.

## 3. Results

### 3.1. Morphological and Structural Characterization of Fe-SC

**Figure 2:**
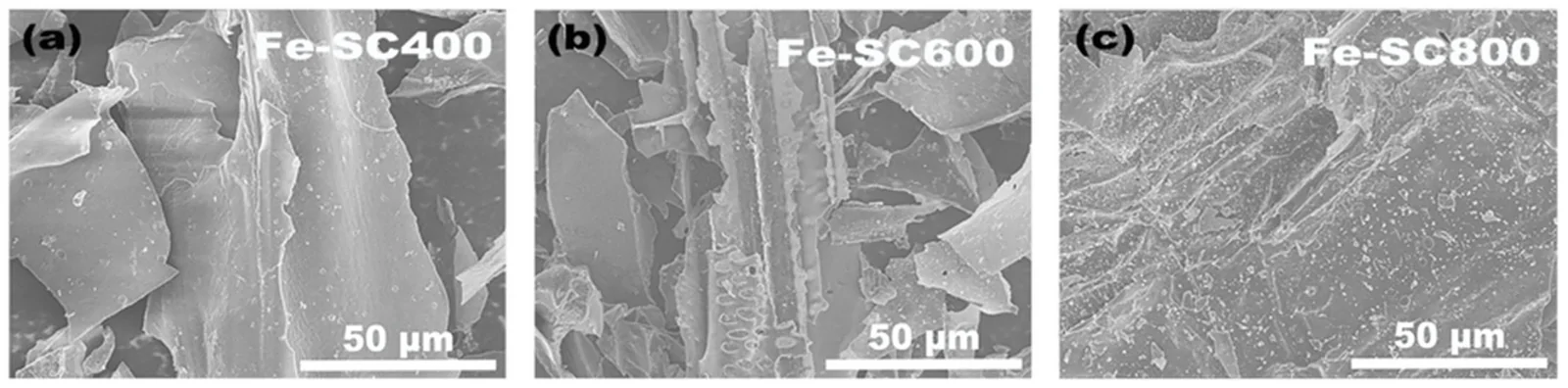
SEM images of Fe-SC. (**a**) SEM image of Fe-SC400. (**b**) SEM image of Fe-SC600. (**c**) SEM image of Fe-SC800.

The morphology and elemental composition of Fe-SC prepared at different temperatures were characterized by SEM. The surface of the Fe-SC had many sheet-like structures along with abundant pores (Figure 2a–c), facilitating iron loading. When the pyrolysis temperature was raised from 400 °C to 800 °C, the Fe-SC exhibited a denser structure with tightly packed granules (Figure 2a–c). Meanwhile, the specific surface area of Fe-SC was characterized using BET (Table S1). For the sample Fe-SC400 pyrolyzed at 400 °C, the specific surface area was 1.17 m^2^/g. In contrast, the specific surface area for Fe-SC600 produced at a higher pyrolysis temperature of 600 °C significantly increased to 217.73 m^2^/g. However, further increasing the pyrolysis temperature to 800 °C for the Fe-SC800 sample resulted in a substantial decrease in the specific surface area to 78.49 m^2^/g. These findings indicated that the relationship between pyrolysis temperature and specific surface area was non-linear, exhibiting an initial increase followed by a decline. This temperature-dependent behavior highlighted the critical role of pyrolysis conditions in Fe-SC. In summary, the pyrolysis-temperature-induced variations in the surface morphology, elemental composition, and specific surface area of Fe-SC are potentially accompanied by the structural phase transformations of the active species.

**Figure 3:**
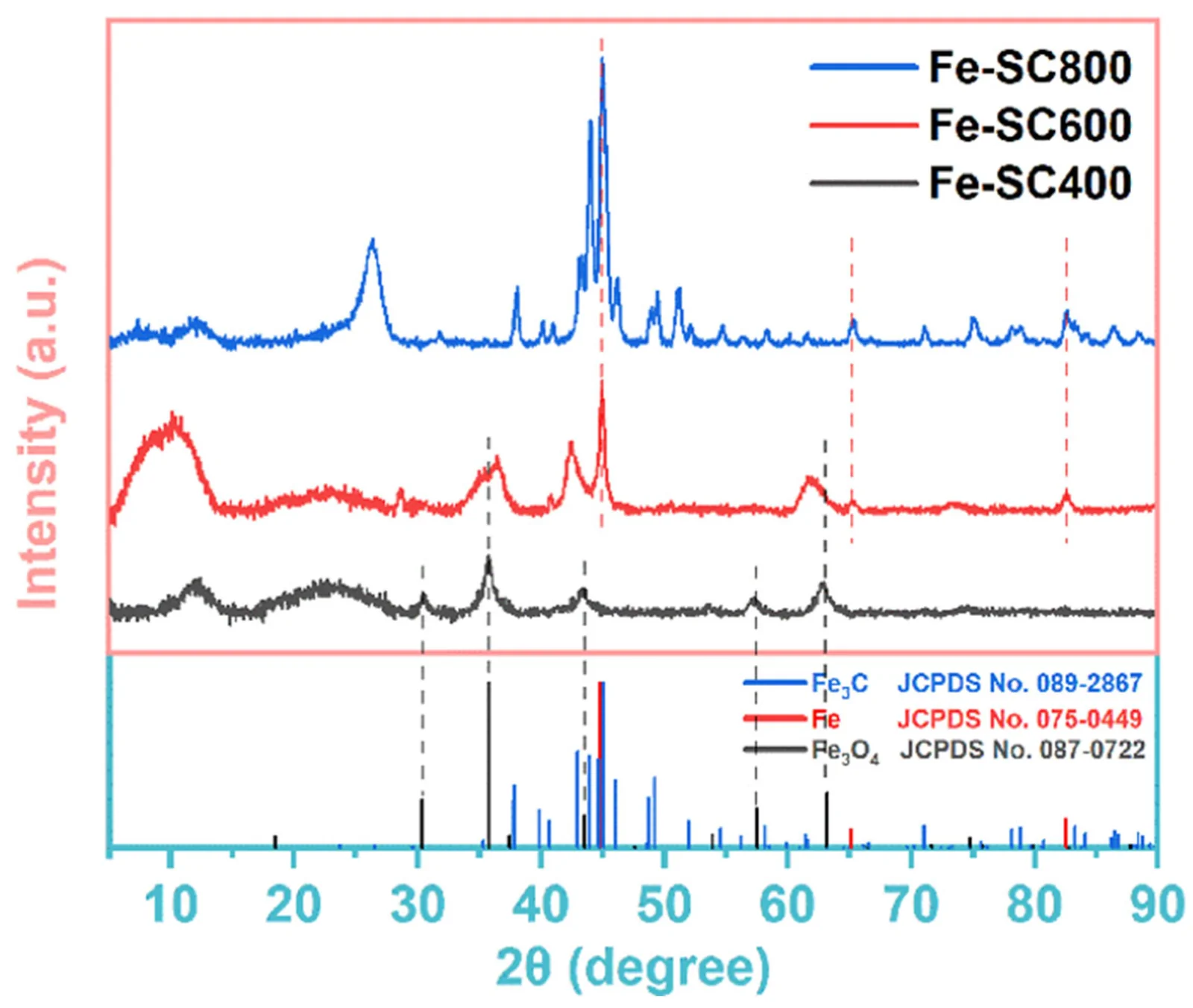
XRD patterns of Fe-SC.

The crystallographic analysis of Fe-SC by XRD revealed significant temperature-induced structural phase transitions (Figure 3). For Fe-SC800, a prominent diffraction peak was observed at a 2θ angle of about 26.30°, which is characteristic of graphitic carbon, indicating that the biomass was converted to graphite at higher pyrolysis temperatures. In contrast, for Fe-SC400, several distinct diffraction peaks corresponding to Fe_3_O_4_ were identified at 2θ angles of about 30.41°, 35.71°, 42.49°, 57.45°, and 63.01°. These peaks indicated the presence of Fe_3_O_4_ as the predominant iron phase at this lower pyrolysis temperature. However, as the pyrolysis temperature was increased to 600 °C, the intensity of these Fe_3_O_4_ peaks diminished, indicating a reduction in the crystalline Fe_3_O_4_ content. By 800 °C, Fe_3_O_4_-related peaks were no longer clearly discernible, suggesting that Fe_3_O_4_ was largely transformed or reduced to other iron phases. Furthermore, new diffraction peaks corresponding to Fe were detected at 2θ angles of about 44.88°, 65.23°, and 82.61° in both Fe-SC600 and Fe-SC800, with higher peak intensities observed for Fe-SC800. The XRD patterns suggest a possible reduction tendency from Fe_3_O_4_ to metallic Fe with increasing pyrolysis temperature. However, the coexistence of residual Fe_3_O_4_ and other Fe species cannot be excluded. Additionally, the emergence of Fe_3_C was observed at 800 °C, marking a further transformation of the iron phases. The combination of a series of phase transitions from Fe_3_O_4_ to Fe and finally to Fe_3_C with the formation of graphitic carbon highlighted the significant impact of pyrolysis temperature on both the iron phase and carbon structure in Fe-SC. However, it should be emphasized that these phase assignments are based mainly on XRD peak identification. A fully quantitative phase analysis was not performed in this study. Because Fe_3_O_4_, metallic Fe, and Fe_3_C may coexist and their diffraction peaks may overlap, the proposed Fe phase evolution should be regarded as tentative rather than conclusive. Active adsorption species on the surface were an important factor that determined the efficacy of adsorbent materials [31,32,33], and the pyrolysis temperature can change the type and structure of the active adsorbent species, which plays a key role in the adsorbent performance.

### 3.2. Sb(III) Adsorption Behavior

#### 3.2.1. Effect of Adsorbent Dose and Adsorption Isotherms

**Figure 4:**
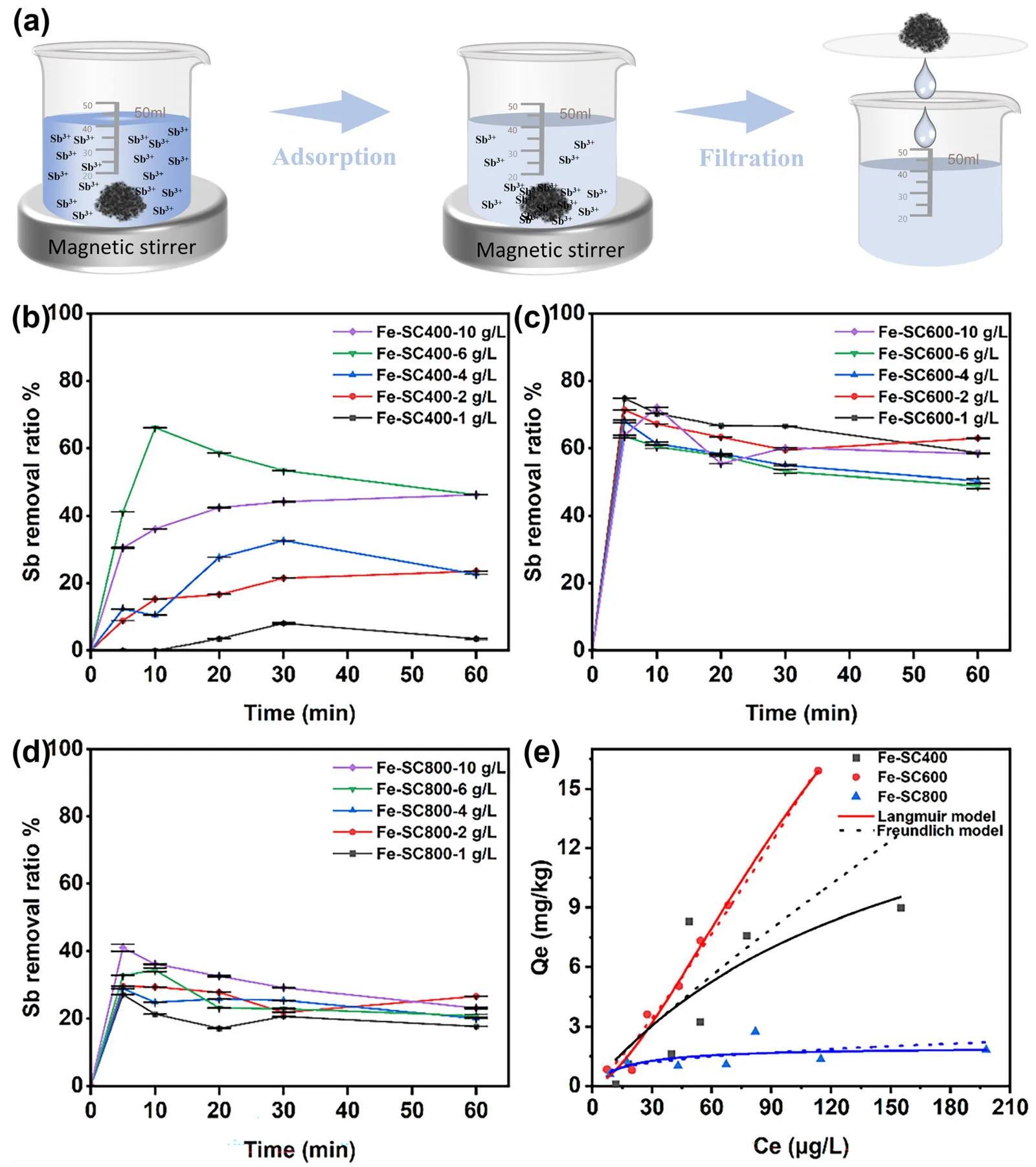
Effect of Fe-SC on Sb(III) adsorption and adsorption isotherms on Fe-SC. (**a**) Digital photographs showing the contact between Fe-SC powder and the Sb(III)-containing aqueous solution and the subsequent filtration process. (**b**–**d**) The influence of Fe-SC400, Fe-SC600, and Fe-SC800 on the adsorption of Sb(III). (**e**) Adsorption isotherms on Fe-SC.

The effects of the dosage of Fe-SC prepared at different temperatures on the adsorption and removal of Sb(III) were systematically analyzed (Figure 4). The findings revealed that the Sb(III) removal efficiency by Fe-SC400 exhibited a positive correlation with dosage up to 6 g/L, where the removal rate peaked at approximately 70%. However, a further increase in dosage to 10 g/L resulted in a decline in the removal rate, suggesting an optimal dosage threshold (Figure 4b). For Fe-SC600, the removal rate of Sb(III) was less sensitive to changes in dosage, consistently achieving a high removal rate (60–80%) at different dosages (Figure 4c). Notably, the removal rate of Fe-SC600 reached a maximum of nearly 80% at the lowest dosage (1 g/L), suggesting that Fe-SC600 maintains a high adsorption capacity even at the lowest dosage (Figure 4c). Conversely, Fe-SC800 demonstrated limited effectiveness in Sb(III) removal across all tested dosages, with the maximum removal rate not exceeding 45%, indicating the low efficiency of Fe-SC800 as an Sb(III) adsorbent under the studied conditions (Figure 4d). The observed trend in the removal rate of Sb(III) by Fe-SC with different dosages indicated that increasing the adsorbent dosage does not necessarily improve the removal efficiency. At higher adsorbent doses, the increase in the number of adsorption sites and the lack of ion concentration led to the agglomeration of the adsorbent, which led to a decrease in the removal rate [34]. Furthermore, Fe-SC achieved its maximum adsorption capacity within the first 5 min, highlighting its rapid adsorption capability. Among the Fe-SC variants tested, Fe-SC600 exhibited the most favorable performance, achieving the highest removal rate of Sb(III) with a minimum dosage requirement of 1 g/L.

Adsorption isotherms were commonly used to evaluate the adsorption properties of materials and were essential for identifying the type of adsorption and determining the adsorption capacity of the adsorbent [35]. Langmuir and Freundlich adsorption isotherms were used to fit the experimental data (Figure 4e and Table S2). According to the Langmuir isotherm, the maximum adsorption capacities of Sb(III) on Fe-SC400, Fe-SC600, and Fe-SC800 were 0.0195, 0.0410, and 0.0020 mg/g, respectively (Table S2). Indeed, even the maximum adsorption capacity of Fe-SC600 of 0.0410 mg/g is significantly lower than the reported value [10,17]. However, this difference mainly arises from the non-comparable experimental conditions rather than from the intrinsic adsorption performance of Fe-SC600. In this study, the isotherm experiments were conducted at Sb(III) concentrations of 20–200 μg/L with an adsorbent dose of 6 g/L. This concentration range was selected to better mimic actual Sb-contaminated water, because mg/L-level concentrations are generally far too high to be environmentally relevant. Such a low initial concentration combined with a relatively high adsorbent dose inherently limits the achievable adsorption capacity. In contrast, related research [10,17] used Sb(III) concentrations of 30–50 mg/L and 50–150 mg/L, respectively, which are two to three orders of magnitude higher. Because adsorption capacity strongly depends on the concentration and adsorbent dose, these values are not directly comparable. This study used a high adsorbent dose and a low initial concentration range, which limits the maximum achievable adsorption capacity and prevents direct benchmarking against high-concentration studies. The main focus of this work is to elucidate the effect of temperature-induced Fe phase transitions on Sb(III) adsorption behavior rather than to maximize the adsorption capacity.

Moreover, it is worth noting that the adsorption isotherms were plotted based on the adsorbent dose of 6 g/L, which resulted in a smaller adsorption capacity for Fe-SC600. The 6 g/L adsorbent dose was chosen to plot the adsorption isotherms by integrating the relationship between the adsorbent dose and Sb removal for Fe-SC400, Fe-SC600, and Fe-SC800 (Figure 4b–d), which enabled the comparison of the effect of the temperature-induced structural phase transitions on the adsorbent adsorption performance at the same level. Meanwhile, excellent adsorption performance was obtained for Fe-SC600 at even a very small adsorbent dose (1 g/L) (Figure 4c). It can be expected that excellent adsorption performance can be maintained for Fe-SC600 even if the adsorbent dosage is further reduced until a threshold value. Therefore, using an adsorbent dose of 1 g/L or less to plot the adsorption isotherms would greatly increase the adsorption capacity of Fe-SC600. The maximum adsorption capacity of 0.0410 mg/g of Fe-SC600 based on the adsorbent dose of 6 g/L was designed to compare the effects of structural phase transitions induced by different temperatures on the adsorption performance of the adsorbent, rather than the maximum adsorption capacity of the Fe-SC600 adsorbent.

The value of 1/n in the Freundlich isotherm model indicates the difficulty of adsorption; the occurrence of adsorption is favorable when 0 < 1/n < 1 and unfavorable when 1/n > 1 (Table S2) [36]. Therefore, the 1/n value (0.86) of Fe-SC600 was between 0 and 1 for favorable adsorption, while the 1/n values of Fe-SC400 (1.15) and Fe-SC800 (2.99) were greater than 1 for unfavorable adsorption (Table S2). The adsorption performance of Fe-SC400 and Fe-SC800 was more consistent with the Langmuir model, with higher R^2^ (0.63, 0.24) values than the Freundlich model (0.45, 0.17) (Table S2). In general, the Langmuir model can describe the monolayer adsorption of adsorbate on a homogeneous surface, including chemical and physical adsorption [37]. In contrast, the Freundlich model could adequately interpret the sorption behavior of Sb(III) on Fe-SC600 based on the Freundlich R^2^ value (0.98) is higher than the Langmuir R^2^ value (0.91) (Table S2). The Freundlich model can simulate heterogeneous multilayer chemisorption [38]. Overall, the differences between Fe-SC adsorption isotherms might be attributed to the differences in adsorption active phases, surface functional groups, and pore structures.

#### 3.2.2. Adsorption Kinetics

**Figure 5:**
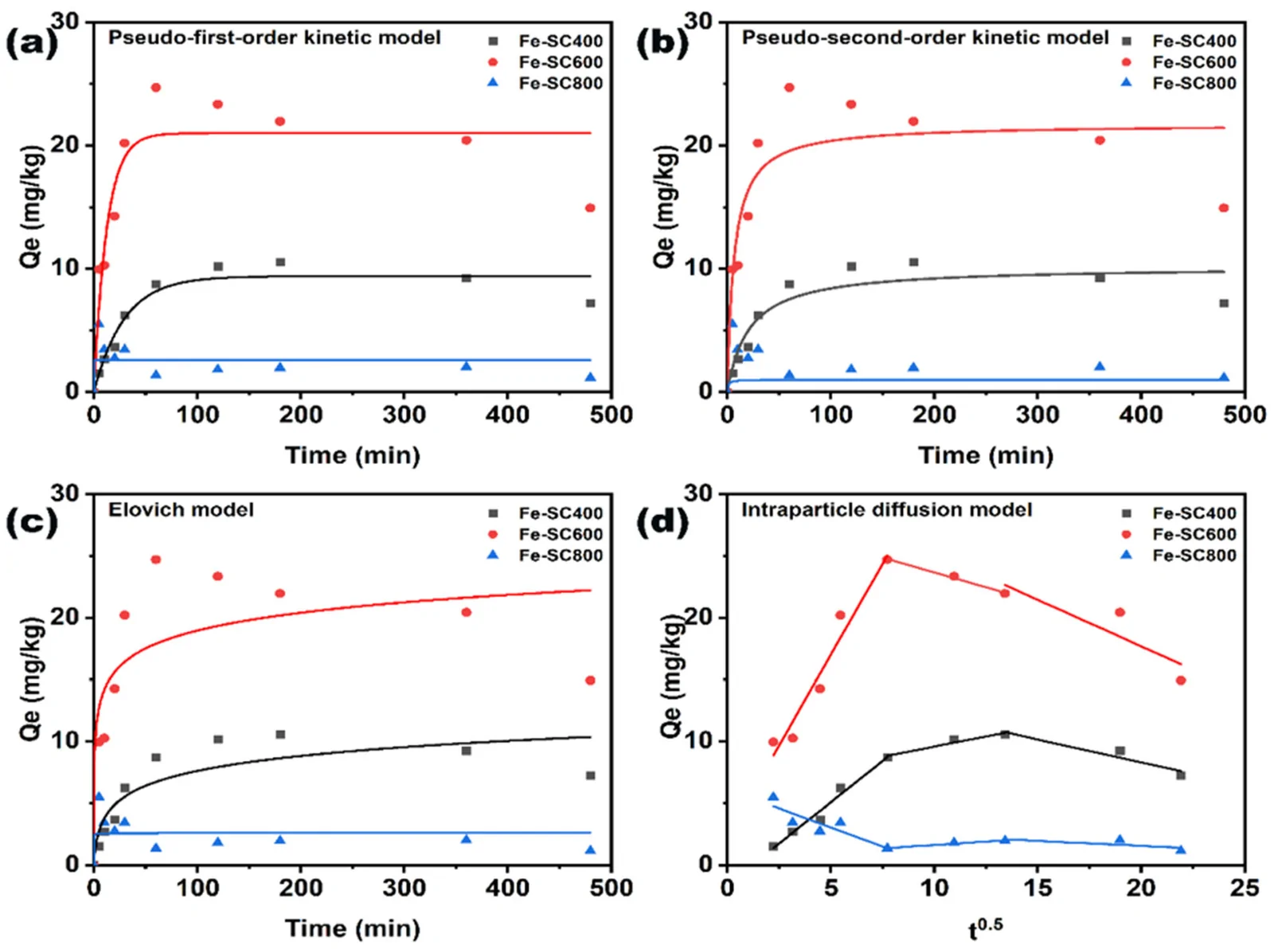
Adsorption kinetics of Fe-SC for Sb(III) adsorption. (**a**) Pseudo-first-order kinetic model. (**b**) Pseudo-second-order kinetic model. (**c**) Elovich model. (**d**) Intragranular diffusion model.

Adsorption kinetics are often used to estimate adsorption rates, which are a key index in determining the adsorption performance of adsorbents [8]. The adsorption kinetics of Sb(III) on the adsorbent were simulated by using mathematical models such as the Pseudo-first-order kinetic model, Pseudo-second-order kinetic model, Elovich model, and Intraparticle diffusion model (Figure 5). The results indicated that the adsorption correlation coefficient R^2^ of the Pseudo-second-order kinetic model was larger than that of the Pseudo-first-order kinetic model and the Elovich model, suggesting that the Pseudo-second-order model provided the best fit (Table S3). Therefore, the mechanism of Fe-SC adsorption of Sb(III) was related to chemical reactions such as redox reactions [39]. The adsorption capacities of Fe-SC400, Fe-SC600, and Fe-SC800 calculated using a Pseudo-second-order model (6.67, 17.79, and 1.33 mg/kg) were closer to experimental data (7.22, 14.93, 1.17 mg/kg) than those calculated using a Pseudo-first-order model (9.39, 20.99, 1.74 mg/kg) (Table S3). In addition, all curves of Fe-SC adsorption of Sb(III) were multilinear in the fitting of the intragranular diffusion model, and the intercept was not zero, indicating that intragranular diffusion was not the only step in the adsorption of Sb(III) by Fe-SC. These three regions of the intra-particle diffusion model correspond to the external mass transfer stage, the intra-particle diffusion stage, and the adsorption equilibrium stage in the Sb(III) adsorption process, respectively [40]. Meanwhile, Fe-SC rapidly adsorbed Sb(III) for the first 5 min, and the reaction reached equilibrium after about 90 min (Figure 5). In summary, the adsorption of Sb(III) by Fe-SC was mainly through chemical bonding, which was related to the surface charge of biochar.

### 3.3. Effect of Initial pH and Coexisting Ions

**Figure 6:**
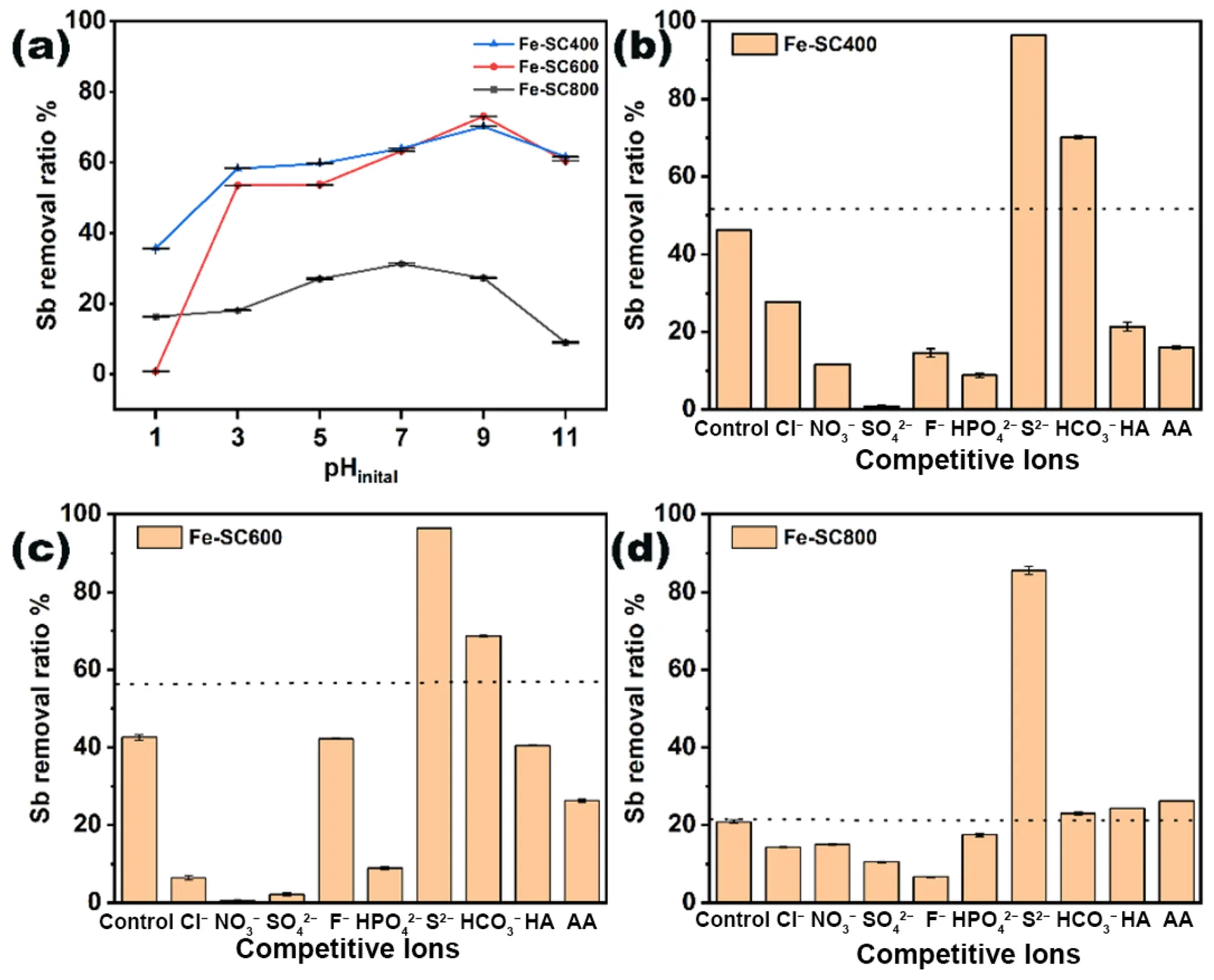
Effect of initial pH and coexisting ions. (**a**) Effect of solution pH on the removal ability of Fe-SC on Sb(III). (**b**–**d**) Effect of coexisting ions on the removal of Sb(III) from water by Fe-SC400, Fe-SC600, and Fe-SC800.

To further explore the interaction between Fe-SC and Sb(III), the effect of the initial solution pH (1.0–11.0) on Sb(III) removal was investigated (Figure 6a). At pH < 3, the increased concentrations of H^+^ may compete with Sb(III) for adsorption sites and suppress the deprotonation of surface hydroxyl groups, resulting in lower Sb(III) adsorption. As the pH increased from 1.0 to 3.0, the adsorption efficiency increased, which may be related to the deprotonation of surface functional groups and the resulting increase in negative surface charge of the Fe-SC and enhancing its ability to bind Sb [41,42], although electrostatic effects alone cannot fully explain the interaction. In the pH range of 3.0–9.0, the adsorption capacity of Fe-SC for Sb(III) remained almost unchanged. Sb(OH)_3_ is an amphoteric hydroxide, and its charge state varies with pH. Therefore, electrostatic interactions may contribute to Sb(III) uptake to some extent; however, they alone cannot fully explain the observed pH-dependent adsorption behaviour. Instead, the adsorption of Sb(III) may proceed through surface complexation with Fe-OH sites and/or ligand exchange. When the pH increased to 11.0, the adsorption capacity decreased slightly. This may be attributed to the possible formation of anionic Sb(OH)_4_^−^ and competition between OH^−^ and Sb species for adsorption sites [43], which may weaken Sb(III) binding. It should be noted that the pHpzc and surface charge of Fe-SC were not quantitatively determined in this study; consequently, the proposed mechanisms are discussed tentatively. In conclusion, Fe-SC exhibited Sb(III) adsorption over a wide pH range. Because most antimony-containing wastewater is neutral or weakly alkaline, Fe-SC may have broad pH adaptability for the practical treatment of antimony-containing wastewater.

Coexisting substances may affect Sb(III) removal by competing for adsorption sites or changing solution chemistry. Therefore, the effects of common ions in antimony-containing wastewater, including chloride (Cl^−^), nitrate (NO_3_^−^), sulfate (SO_4_^2−^), hydrogen phosphate (HPO_4_^2−^), fluoride (F^−^), sulfide (S^2−^), bicarbonate (HCO_3_^−^), humic acid (HA, natural dissolved organic compounds), and acetic acid (AA, low-molecular-weight organic acid), on the Sb(III) adsorption capacity of Fe-SC were explored. S^2−^ and HCO_3_^−^ enhanced Sb(III) removal under the tested conditions, whereas the other ions inhibited Sb(III) adsorption by Fe-SC (Figure 6b–d). Coexisting anions may affect Sb(III) adsorption through the formation of precipitates or complexes, competing for adsorption sites, and other interactions, which collectively promote or reduce the adsorption efficiency of Fe-SC. It should be noted that the enhanced Sb removal in the presence of S^2−^ may involve both surface adsorption and possible precipitation of insoluble Sb-S phases. Because a control experiment without adsorbent was not performed, the relative contribution of adsorption and precipitation cannot be distinguished from the present data. Future work will include this control to clarify the mechanism.

### 3.4. Adsorption Mechanism

**Figure 7:**
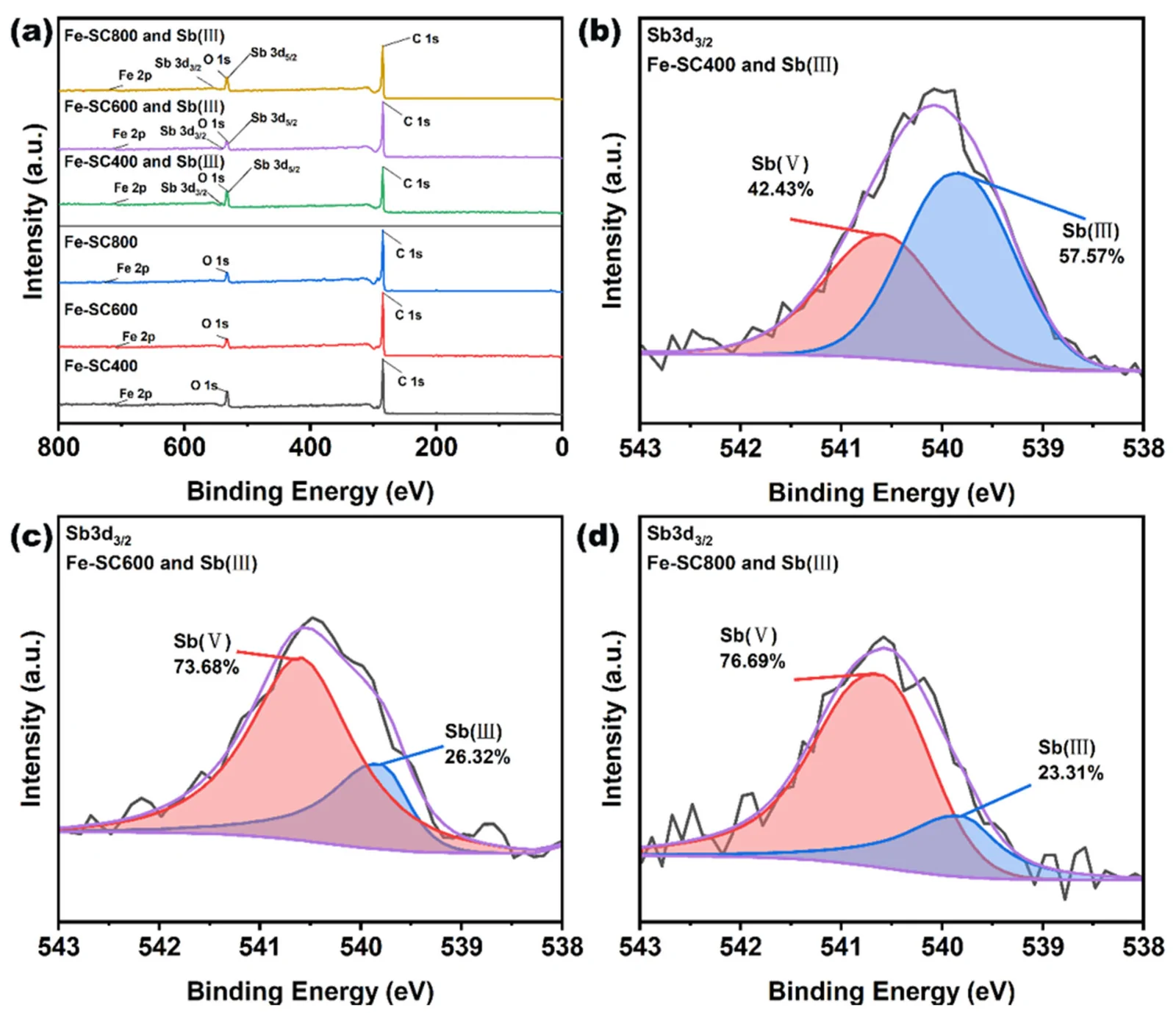
X-ray photoelectron spectroscopy (XPS) of Fe-SC and Sb-loaded Fe-SC. (**a**) XPS full-scan spectra of Fe-SC400, Fe-SC600, and Fe-SC800 before and after Sb (III) adsorption. (**b**) Sb 3d_3/2_ XPS spectra of Fe-SC400 after adsorption of Sb(III). (**c**) Sb 3d_3/2_ XPS spectra of Fe-SC600 after adsorption of Sb(III). (**d**) Sb 3d_3/2_ XPS spectra of Fe-SC800 after adsorption of Sb(III).

The XPS spectra of Fe-SC before and after Sb(III) adsorption are shown in Figure 7a. Because the Sb 3d_5/2_ peak partially overlaps with the O 1s peak, only the Sb 3d_3/2_ peak was fitted. The binding energies of Sb 3d_3/2_ for Sb(III) and Sb(V) were observed at 539.8 eV and 540.6 eV, respectively. After Sb(III) adsorption, both Sb(III) and Sb(V) were present on the Fe-SC surface. Based on the fitted Sb 3d3/2 peak areas, the apparent surface Sb(V) fractions were 42.43%, 73.68%, and 76.69% for Fe-SC400, Fe-SC600, and Fe-SC800, respectively (Figure 7b–d). These values are considered semi-quantitative surface Sb species proportions rather than absolute oxidation efficiencies. It should be noted that XPS is a surface-sensitive and ex situ technique, and sample exposure to air during preparation and transfer may affect the observed Sb oxidation state. Therefore, the XPS data alone cannot conclusively establish the oxidation mechanism. Metallic Fe is a reducing species and cannot directly serve as the electron acceptor for Sb(III) oxidation. Possible electron acceptors may include dissolved oxygen, Fe(III) species, or reactive oxygen species generated during Fe redox cycling. However, the changes in Fe oxidation state before and after Sb adsorption, the role of dissolved oxygen, the formation of reactive oxygen species, and Fe leaching were not determined in this study. Consequently, the partial conversion of Sb(III) to Sb(V) observed by XPS should be regarded as a tentative surface process rather than a confirmed function of Fe-SC. The exact role of Fe species in this process remains to be clarified.

In summary, the adsorption performance of Fe-SC for Sb (III) is closely related to the changes in iron-containing phases (Fe_3_O_4_, Fe, and Fe_3_C), changes in specific surface area, and hydrophobicity caused by graphitization. As the pyrolysis temperature increases, Fe-SC undergoes a significant structural phase transition. The Fe-SC primarily consisted of the Fe_3_O_4_ crystalline phase at 400 °C. As the pyrolysis temperature increased to 600 °C, the Fe_3_O_4_ crystalline phase gradually converted into the Fe crystalline phase. Further increasing the temperature to 800 °C led to the formation of the Fe_3_C crystalline phase, while the biochar was significantly graphitized. Fe-SC800 showed greater graphitic ordering and lower Sb(III) removal than Fe-SC600. Meanwhile, the specific surface area initially increased with increasing temperature, thus improving the adsorption capacity. Fe-SC600 had the highest specific surface area due to the optimal pyrolysis temperature (600 °C), resulting in superior Sb(III) removal efficiency. However, the specific surface area decreased at 800 °C due to excessive graphitization, which reduced the availability of adsorption sites.

**Figure 8:**
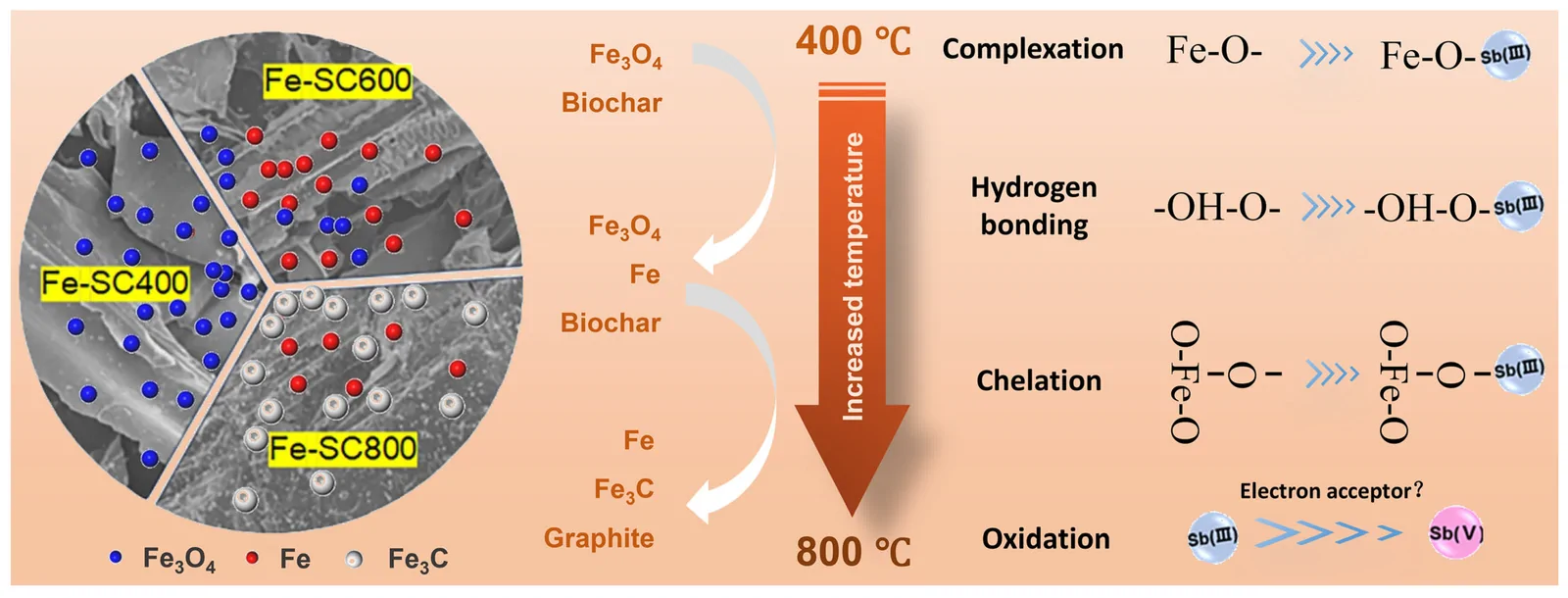
Proposed mechanism of Fe SC for Sb(III) adsorption.

In conclusion, the temperature-induced structural phase transitions and the degree of graphitization were crucial in determining the pollutant adsorption capabilities of Fe-SC. The removal of Sb(III) by Fe-SC involves a multi-step process (Figure 8). Initially, Sb(III) species diffuse from the bulk aqueous solution to the external surface of the Fe-SC powder. Subsequently, Sb(III) may interact with the surface functional groups and Fe-containing sites through possible pathways, including hydrogen bonding, surface complexation, and chelation. These pathways are represented by dashed arrows (proposed mechanism) in Figure 8. The relative contribution of these interactions depends on the pyrolysis temperature, which controls the phase composition (Fe_3_O_4_, metallic Fe, and Fe_3_C), the specific surface area, and the degree of graphitization. The structural properties of Fe-SC600 were optimally balanced to exhibit the highest Sb(III) removal efficiency. In addition, XPS analysis suggests that a fraction of Sb(III) may be converted to Sb(V) on the Fe-SC surface. However, the electron acceptor and the exact redox pathway were not identified in this study. Finally, the Sb-loaded Fe-SC powder is separated from the aqueous phase by filtration (Figure 4a). These findings underscore the importance of optimizing pyrolysis conditions to tailor the adsorption performance of Fe-SC for specific environmental applications.

Beyond pyrolysis conditions, the selection of the biomass precursor also plays an important role in the sustainable and scalable production of biochar. In this study, sugarcane bagasse was selected because sugarcane is widely cultivated with large annual production, and bagasse is a major byproduct of the sugar industry, making it an abundant, low-cost, and readily available feedstock. Compared with other biomass sources such as spent coffee grounds, coconut shells, rice husks, and walnut shells, sugarcane bagasse offers clear advantages in supply volume and regional availability. These features make it suitable for large-scale preparation of biochar for environmental applications. The Sb(III) removal performance of Fe-SC has been compared with previously reported biochar-based materials. A recent systematic review by K. Abhishek et al. reported Sb(III) removal efficiencies of 53–100% for biochar-based materials [44]. The Sb(III) removal efficiency of Fe-SC600 in this study is nearly 80%, which is in the middle range of these reported values. It should be emphasized that the primary focus of this work is not to maximize the adsorption capacity, but to elucidate how the temperature-induced Fe phase evolution (Fe_3_O_4_ - metallic Fe - Fe_3_C) governs the Sb(III) adsorption behavior. Utilizing such renewable biomass for functional carbon materials is consistent with the sustainable development of bio-based composites and nanocomposites [45].

It should be noted that adsorbent reusability/regeneration tests, real wastewater experiments, Fe 2p XPS analysis before/after adsorption, and Fe leaching measurements were not performed in this study. Consequently, the practical applicability, long-term stability, and potential Fe release of Fe-SC cannot be evaluated from the present data. These aspects should be addressed in future work to further assess the feasibility of Fe-SC for real wastewater treatment.

## 4. Conclusions

This study examined how pyrolysis temperature affects the structure and Sb(III) removal performance of iron-modified sugarcane bagasse biochar. Among the materials prepared at 400, 600, and 800 °C, Fe-SC600 had the highest specific surface area (217.73 m^2^/g) and achieved the greatest Sb(III) removal, approaching 80% under the tested conditions. Pyrolysis temperature was identified as the key factor controlling the Fe phase composition, graphitization degree, and specific surface area of Fe-SC. XRD patterns suggested changes in iron-containing phases with increasing temperature, although the phases were not quantified and may coexist. Sb(III) removal remained relatively stable over a broad pH range of 3–9. S_2_^−^ and HCO_3_^−^ increased Sb removal, but precipitation may have contributed to the result observed with sulfide. These findings suggest that controlling pyrolysis temperature could help develop sugarcane bagasse biochar for treating Sb-contaminated water. To assess this potential, future studies should test the material in real wastewater, evaluate its regeneration and repeated use, measure Fe leaching, and distinguish adsorption from precipitation in the presence of sulfide.

## Data Availability

The raw data supporting the conclusions of this article will be made available by the authors on request.

## Supplementary Materials

The following supporting information can be downloaded at: https://www.mdpi.com/article/10.3390/nano16201288/s1, Text S1: Adsorption isotherms; Text S2: Adsorption kinetics; Table S1: Specific surface area of Fe-SC at three pyrolysis temperatures; Table S2: Adsorption isotherm parameters on Fe-SC; Table S3: Adsorption kinetic parameters on Fe-SC.
