# Supplementary material for: Temperature-Induced Structural Phase Transitions in Nano-Fe-Modified Sustainable Biochar for Efficient Sb(III) Adsorption
Source: Nanomaterials (Basel). 2026 Oct 11;16(20):1288. doi: 10.3390/nano16201288 (PMC13707491; doi:10.3390/nano16201288)
Supplement: Supplementary file 1 [file nanomaterials-16-01288-s001.zip › nanomaterials-4532841 - Supplementary Materials.pdf]

Temperature-induced structural phase transitions in nano-Fe modified sustainable biochar for efficient Sb(III) adsorption

Lei Wang [1, 2, *], Bo Song [3], Qi Wang [1, 4], Gaoyani Ku [1, 2], Chen Chen [5], Zhibin Wang [2], Jafar Ali [6, 7], Evgeni M. Glebov [8, 9], Xuliang Zhuang [1, 2, 10, *]

[1] Laboratory of Environmental Biotechnology, Research Center for Eco-Environmental Sciences, Chinese Academy of Sciences, Beijing 100085, China

[2] College of Resources and Environment, University of Chinese Academy of Sciences, Beijing 100049, China

[3] Institute of Fungus Resources, Key Laboratory of Plant Resource Conservation and Germplasm Inno-vation in Mountainous Region (Ministry of Education), College of Life Sciences, Guizhou University, Guiyang 550025, Guizhou Province, China

[4] Henan Institute of Advanced Technology, Zhengzhou University, Zhengzhou 450003, China

[5] Hubei Key Laboratory for Processing and Transformation of Agricultural Products, College of Food Science & Engineering, Wuhan Polytechnic University, Wuhan 430023, China

[6] Institute of Sustainable Energy (ISE), Universiti Tenaga Nasional (UNITEN), Malaysia

[7] Department of Water Quality Management, Karlsruhe Institute of Technology (KIT), Gotthard-Franz-Str. 3, 76131, Karlsruhe, Germany

[8] Voevodsky Institute of Chemical Kinetics and Combustion, Siberian Branch of the Russian Academy of Sciences, 3 Institutskaya Str., 630090 Novosibirsk, Russian Federation

[9] Novosibirsk State University, 2 Pirogova Str., 630090 Novosibirsk, Russian Federation

[10] State Key Laboratory of Tibetan Plateau Earth System, Environment and Resources (TPESER), Institute of Tibetan Plateau Research, Chinese Academy of Sciences, Beijing 100101, China

*** Corresponding Author.**

E-mail addresses:

Lei Wang: leiwang@rcees.ac.cn; Xuliang Zhuang: xlzhuang@rcees.ac.cn

Text S1. Adsorption isotherms

Langmuir and Freundlich isotherms were selected in the study:

Langmuir model:

$$q_e = \frac{Q_{max}K_L C_e}{1+K_L C_e} \quad \text{(S1)}$$

Freundlich model:

$$q_e = K_F C_e^{\frac{1}{n}} \quad \text{(S2)}$$

Where q_e ($mg \cdot kg^{-1}$) and Q_{max} ($mg \cdot kg^{-1}$) are the adsorption capacity for Sb(III) at the equilibrium time and the maximum adsorption capacity, respectively; Ce ($\mu g \cdot L^{-1}$) is equilibrium concentration; K_F ($L \cdot mg^{-1}$) and K_L ($L \cdot mg^{-1}$) are the Freundlich and Langmuir characteristic constant, respectively; n is a characteristic constant characterizing the properties of adsorption force (when $1/n < 1$, the adsorbate is easy to be adsorbed; when $1/n > 1$, the adsorbate is difficult to be adsorbed).

Text S2. Adsorption kinetics

Adsorption kinetics and isotherms are often used to evaluate the adsorption performance and process of biochar. Preliminary studies have shown that Fe-SC adsorbents can rapidly adsorb Sb(III). To describe the adsorption rate, the adsorption experimental data of Sb(III) on Fe-SC400, Fe-SC600, and Fe-SC800 were fitted using kinetic equations such as Pseudo-first-order and Pseudo-second-order models, Elovich model, and Intragranular diffusion models. The equation forms are as follows:

Pseudo-first-order kinetic model:

$$\ln(q_e + q_t) = lnq_e - k_1 t \qquad \text{(S3)}$$

Pseudo-second-order kinetic model:

$$\frac{t}{q_t} = \frac{1}{k_2 {q_e}^2} + \frac{t}{q_e} \qquad \text{(S4)}$$

Elovich model:

$$q_t = \frac{1}{\beta} ln\alpha\beta + \frac{1}{\beta} lnt \qquad \text{(S5)}$$

Intraparticle diffusion model:

$$q_t = k_i t^{\frac{1}{2}} + C \qquad \text{(S6)}$$

Where q_e ($mg{\cdot}kg^{-1}$) and q_t ($mg{\cdot}kg^{-1}$) are the adsorbed amount of Sb(III) on the adsorbent at equilibrium and at different times, respectively; k_1 ($mg{\cdot}kg^{-1}{\cdot}min^{-1}$) and k_2 ($mg{\cdot}kg^{-1}{\cdot}min^{-1}$) are the rate constants of the pseudo-first-order and pseudo-second-order models, respectively; α ($mg{\cdot}kg^{-1}{\cdot}min^{-1}$) is the sorption kinetic at the beginning and β (kg mg^{-1}) is the Elovich model constant; k_i ($mg{\cdot}kg^{-1}{\cdot}h^{1/2}$) is the intra-particle diffusion rate constant and C ($mg{\cdot}kg^{-1}$) is the intercept of the diffusion equation within the particle.

Table S1. Specific surface area of Fe-SC at three pyrolysis temperatures.

Biochar	S_{BET} ($m^2 \cdot g^{-1}$)
Fe-SC400	1.17
Fe-SC600	217.73
Fe-SC800	78.49

Table S2. Adsorption isotherm parameters on Fe-SC.

System	Langmuir model			Freundlich model		
	Q_{max} (mg·g^{-1})	K_L (L·mg^{-1})	R^2	1/n	K_F (L·mg^{-1})	R^2
Fe-SC400	0.0195	0.006	0.63	1.15	0.16	0.45
Fe-SC600	0.0410	0.005	0.91	0.86	0.07	0.98
Fe-SC800	0.0020	0.06	0.24	2.99	0.38	0.17

Table S3. Adsorption kinetic parameters on Fe-SC.

System	Pseudo-first-order kinetic model			Pseudo-second-order kinetic model			Elovich model		
	q_e (mg·kg^{-1})	k_1	R^2	q_e (mg·kg^{-1})	k_2	R^2	α	β	R^2
Fe-SC400	9.39	0.033	0.35	6.67	1.49	0.96	1.77	-0.5	0.71
Fe-SC600	20.99	0.079	0.93	17.79	-0.0091	0.98	-1.71	15.95	0.92
Fe-SC800	1.74	0.028	0.17	1.33	-0.0373	0.91	-0.72	5.54	0.72

System	Intraparticle diffusion model								
	Phase one			Phase two			Phase three		
	k_{i1}	C_1	R^2	k_{i2}	C_2	R^2	k_{i3}	C_3	R^2
Fe-SC400	1.34	-1.6	0.98	0.33	6.31	0.94	-0.37	15.73	0.91
Fe-SC600	0.34	9.19	0.94	-0.48	28.5	0.99	-0.75	32.75	0.78
Fe-SC800	-0.62	6.14	0.78	0.11	0.55	0.96	-0.08	3.16	0.53
